# Clinical Features of Primary Vein Grafts in Free Tissue Transfers

**DOI:** 10.1155/2015/481402

**Published:** 2015-03-12

**Authors:** Mitsuru Nemoto, Kenichi Kumazawa, Eiju Uchinuma, Natsuko Kounoike, Akira Takeda

**Affiliations:** Department of Plastic and Reconstructive Surgery, Kitasato University Hospital, 1-15-1 Kitasato, Minami-ku, Sagamihara, Kanagawa 252-0374, Japan

## Abstract

The outcomes of free tissue transfers combined with vein grafts have been inconsistent, and discussions continue regarding their appropriate use. Of the 142 free tissue transfers that we performed from January 2004 to December 2011, we retrospectively analyzed 15 consecutive patients who underwent free tissue transfers in combination with vein grafts. Etiologies included trauma (8 patients), infection (4), and tumor (3). Types of free tissue transfers were fibula (4), anterolateral thigh (3), groin (3), jejunum (3), latissimus dorsi (1), and dorsal pedis (1). Vein grafts were used for the artery (6), vein (2), or both (7). The donor veins were the saphenous vein (12) and the external jugular vein (3). The mean length of the grafted veins was 10.8 cm (range: 4–18 cm). Even though complications of congestion occurred in 2 patients, these flaps survived by reexploration. The flap success rate was 15 of 15 (100%) of vein grafted free flaps versus 124 of 127 (97.6%) of free flaps not requiring vein grafts. To improve the success rate of free tissue transfers combined with vein grafts, securing healthy recipient vessels, meticulous surgical handling, a reliable vascular anastomosis technique, and strict postoperative monitoring are crucial.

## 1. Introduction

Vein grafts used in combination with free tissue transfers are known to increase the risk of postoperative complications [1, 2]. Oliva [3] reported a failure rate of approximately 20% with this particular combined procedure. Miller et al. [4] stated that free tissue transfers to recipient sites that have been preoperatively exposed to radiation were unsuccessful in approximately 30% of the cases. Conversely, Germann and Steinau [5] reported that the success rate of free flaps combined with vein grafts was 96.7% and the success rate of free flaps without vein grafts was 96.2%, indicating a lack of significant difference between these two methods. Bayramiçli et al. [6] also reported that there were no significant differences in the success rates between free tissue transfers with or without vein grafts. The present study retrospectively analyzed the clinical features of 15 consecutive patients who underwent free tissue transfers with vein grafts to improve the flap success rate.

## 2. Patients and Methods

Using the medical records of all 142 patients who underwent free tissue transfers from January 2004 to December 2011 at our institution, we retrospectively analyzed 15 consecutive patients who underwent free tissue transfers combined with vein grafts. The analyzed items included etiology, timing, anatomic site, type of free tissue, grafted donor vein, length of grafted vein, recipient artery, recipient vein, and postoperative complications. The flap success rate of these 15 patients was compared with that of patients who underwent free tissue transfers without vein grafts using Fisher's exact test.

## 3. Results

The 15 patients were comprised of 13 males and 2 females, with a mean age of 45.8 years (range: 18–84 years) and the mean duration of follow-up was 62 months (range: 26–108 months). The etiologies were open fracture of the lower extremity in 6 patients, lower extremity osteomyelitis in 4 patients, hypopharyngeal carcinoma in 3 patients, and degloving injury in 2 patients. The timing of grafting was immediate in 3 patients, within 7 days in 8 patients, at 6–12 months after injury in 2 patients, and at 12–24 months after injury in 2 patients. Anatomic sites of free tissue transfers were the lower extremity in 10 patients, neck in 3 patients, forearm in 1 patient, and hand in 1 patient. Free tissue was obtained from the fibula in 4 patients, the anterolateral thigh in 3 patients, the groin in 3 patients, the jejunum in 3 patients, the latissimus dorsi in 1 patient, and the dorsal pedis in 1 patient. Grafted donor veins were the saphenous vein in 12 patients and the external jugular vein in 3 patients. The mean length of the grafted veins was 10.8 cm (range: 4–18 cm). Recipient arteries were the posterior tibial artery in 5 patients, the anterior tibial artery in 3 patients, the lingual artery in 3 patients, the popliteal artery in 1 patient, and the radial artery in 1 patient. The recipient veins were the posterior tibial vein in 4 patients, the anterior tibial vein in 2 patients, the saphenous vein in 1 patient, the internal jugular vein in 1 patient, and the cephalic vein in 1 patient. For vein graft-associated complications, congestion developed in 2 patients (1 patient with a degloving injury who underwent a free groin flap and 1 patient with osteomyelitis who underwent a free vascularized fibular flap), for whom additional vein grafts were performed. Though these 2 patients developed partial epidermal necrosis, these flaps survived. In the other 13 patients who received vein grafts, no postoperative complications were encountered, and all flaps survived (Table 1). During the study period, postoperative complications developed in 6 of the 127 patients who underwent a free tissue transfer without vein grafts, and necrosis was found in 3 of these 6 patients. Free flap survival was confirmed in 124 of the 127 patients who did not receive vein grafts. No significant differences in success rates were seen between patients who underwent free tissue transfers with or without vein grafts.

## 4. Case Reports

### 4.1. Case  6

**Figure 1:**
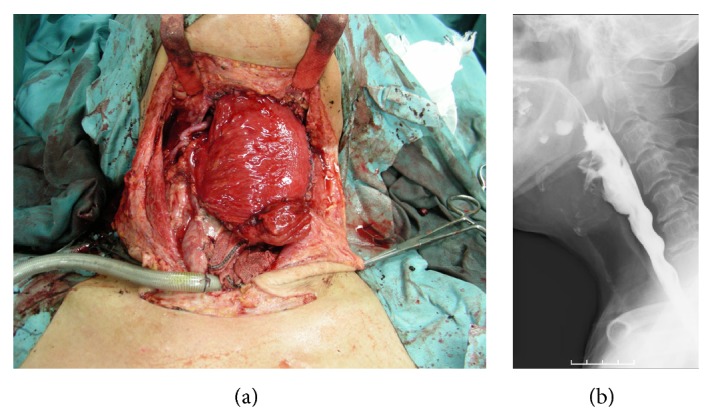
(a) The second jejunal vessels were anastomosed to lingual artery and internal jugular vein with vein grafts. (b) The postoperative X-ray finding of swallowing. There were no leakage and stenosis.

A 71-year-old man with hypopharyngeal carcinoma (staging: T4N2M0) underwent esophageal reconstruction with a free jejunal transfer after undergoing a total pharyngolaryngoesophagectomy and selective neck dissection. With a free jejunal transfer, a second jejunal artery anastomosis to the lingual artery was performed, but thrombosis formation recurred at the anastomotic site. As a result, the free jejunum became a short pedicle, and a vein graft was therefore used to compensate for the insufficient vessel length. An 8 cm long vein harvested from the ipsilateral external jugular vein was interposed between the second jejunal artery and the lingual artery. In addition, a 5 cm external jugular vein was interposed between the second jejunal vein and the internal jugular vein, and the kinking of the vein at the anastomotic site was resolved. After vein graft, thrombosis did not develop, and the free jejunum survived. Neither postoperative leakage nor stenosis occurred at the anastomotic site of the intestinal tract (Figure 1).

### 4.2. Case  11

**Figure 2:**
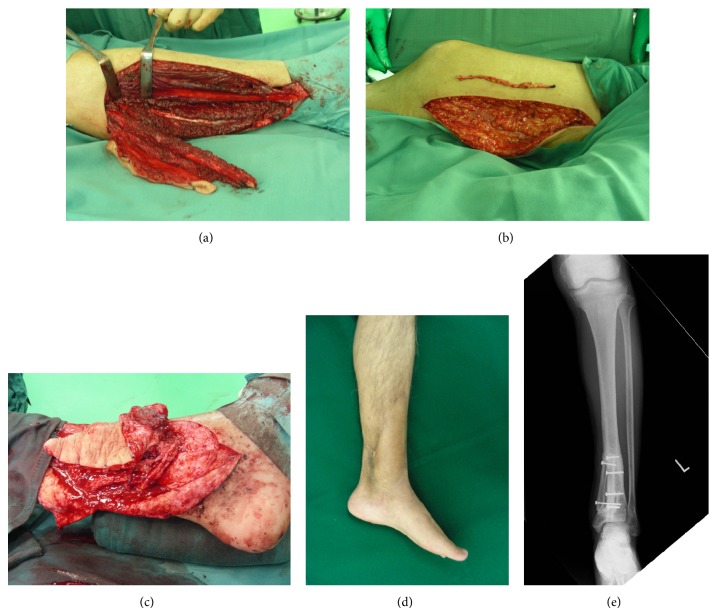
(a) The vascularized fibular flap. (b) The saphenous vein was harvested from right thigh. (c) The saphenous veins were interposed between fibular vessels and posterior tibial vessels. (d) The postoperative view of 50 months after surgery. (e) The osteomyelitis did not recur during the 50 months follow-up period.

A 70-year-old man suffered from an open fracture of the left lower extremity in a traffic accident and underwent plate fixation. Because the fracture site became infected and osteomyelitis developed, the plate was removed, and the site of the bone defect was filled with antibiotic-impregnated cement after debridement. We decided to reconstruct the tibia with a free vascularized fibular flap from the opposite side. Due to the influence of scarring, the appropriate vessels for anastomosis could not be found at the recipient site. The recipient vessel was dissected on the proximal side, away from the zone of injury, and posterior tibial vessels were selected. The saphenous vein on the opposite thigh was harvested for vein grafting and was interposed in the approximately 7 cm length between the fibular vessels and the posterior tibial vessels. Thrombosis did not develop in the free vascularized fibular flap postoperatively and the flap survived. Union of the grafted fibula was obtained 8 months postoperatively. Tibial osteomyelitis has not recurred as of 50 months postoperatively (Figure 2).

### 4.3. Case  14

**Figure 3:**
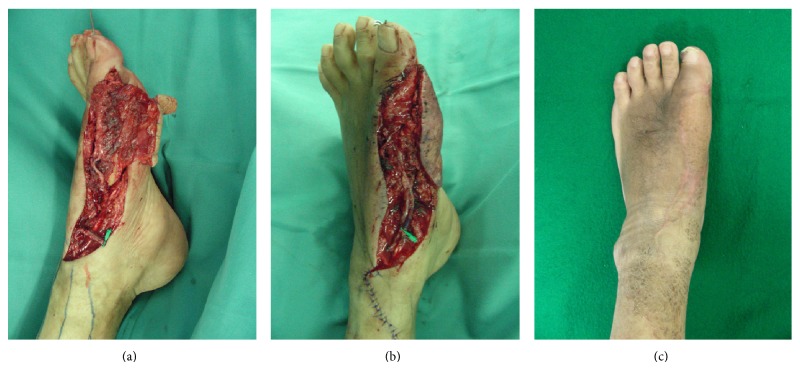
(a) Primary vein grafts were performed for an interarterial and 2 intervenous grafts. (b) Thrombosis was found in the primary vein graft between the comitant vein and the cutaneous vein. (c) Postoperative view 12 months after flap transfer with vein grafts.

A 52-year-old man with diabetes mellitus sustained a deep burn injury to the left foot. The wound progressed to osteomyelitis of the 1st metatarsal bone. The bone defect was filled with antibiotic-impregnated cement after debridement. Six months later, we reconstruct the 1st metatarsal bone with a free vascularized ipsilateral fibular flap. Due to the avoidance of scarring around the 1st metatarsal bone, vein grafts were required to anastomose fibular vessels of the flap and the healthy recipient vessels. The vein grafts were the interposed fibular artery to the dorsal pedis artery and 2 comitant veins to 2 cutaneous veins. The vascularized fibular flap progressed to congestion the next day. We explored the anastomotic site and detected thrombosis in the interposed vein between the comitant vein and the cutaneous vein. The primary grafted vein with thrombosis was resected, and the contralateral small saphenous vein interposed the venous defect between the comitant vein and the healthy cutaneous vein. The congestion of the flap was ameliorated and survived. The osteomyelitis did not recur up to 12 months postoperatively (Figure 3).

## 5. Discussion

Vein grafting is an important technique in reconstructive microsurgery [7–13]. The combined usage of vein grafts with a free tissue transfer is applicable for trauma and neck dissection or when problems exist with the condition of recipient vessels due to radiation, pedicle length, or differences in the caliber of vessels for anastomosis. In animal studies [14, 15], the patency rate of vein grafts is similar to that for normal vascular anastomosis. Despite this, the success rate for a free tissue transfer combined with vein grafts varies between 70% and 95%, depending on the report, and postoperative outcomes have not been consistent, leading to continual discussions on the appropriate use of vein grafting [3–6, 16]. Cheng et al. [17] stated that the failure rate of free tissue transfers combined with vein grafts is approximately 5 times greater than that of free tissue transfers without vein grafts and that the combined usage of vein grafts should be avoided if possible. However, we believe that this combined procedure cannot be avoided and that its usefulness will increase if the reliability of vein grafting can be improved.

In the free tissue transfer combined with vein graft that we performed, 8 patients underwent this operation due to trauma, 4 patients due to infection, and 3 patients due to malignant neck tumor. The soft tissue injury was extensive in degloving injury and open fracture of the lower extremity, and the scarring caused by inflammation was similarly extensive in osteomyelitis. Consequently, free tissue with a long pedicle became necessary to a degree greater than the amount of soft tissue defect and the combined usage of a vein graft became inevitable. In patients with malignant neck tumor, combined usage of a vein graft became necessary due to the extent of malignant tumor infiltration, aging change of recipient vessels, and the influence of radiation.

Bayramiçli et al. [6] succeeded in lower extremity reconstruction using a free tissue transfer with vein grafts in 21 of 22 patients and recommended having a detailed preoperative surgical plan and selecting appropriate recipient vessels under a magnifier when performing vein grafts. Schusterman et al. [13] also reported satisfactory outcomes in planned vein grafts. Planning free tissue transfer combined with vein grafts in advance is important in patients with poor conditions at the recipient sites.

Miller et al. [4] stated coagulation abnormality as well as scarring due to prior operation and radiation as factors affecting outcomes for vein grafts. In our series, postoperative congestion developed in 2 of the 15 patients who received free tissue transfer with vein grafts. One of these patients received a free vascularized fibular flap for osteomyelitis and the other received a groin flap for a degloving injury. Postosteomyelitis scarring and the influence on recipient veins due to the degloving injury were thought to be the causes of congestion. In both patients, we were able to achieve flap survival with early reexploration by dissecting the recipient vessel toward the healthy portion on the proximal side of the initial vessel anastomotic site. Germann and Steinau [5] stated that, although free tissue transfer combined with vein grafts carries a greater complication rate, no significant differences in the ultimate flap survival rate were seen when conducting strict postoperative monitoring and performing early revision when abnormal findings were found. We also salvaged free flaps where congestion occurred with strict monitoring and prompt reexploration, indicating that strict postoperative monitoring is essential for the success of free tissue transfers with vein grafts.

## 6. Conclusion

We obtained comparable clinical outcomes with both free tissue transfer combined with vein grafts and free tissue transfer without vein grafts. To improve the success rate of free tissue transfer combined with vein grafts, securing healthy recipient vessels, meticulous surgical handling, reliable vascular anastomosis techniques, and postoperative monitoring are crucial.

## Figures and Tables

**Table 1:** Patients summary.

Patient number	Age, year	Sex	Etiology	Anatomic site	Type of free tissue	Recipient a.	Recipient v.	Vein graft	Complication	Result	Comments
1	39	M	Open fracture	Lower extremity	Groin	Ant. tibial a.	Ant. tibial v.	Interarterial and intervenous		Successful	
2	27	M	Degloving injury	Hand	Dorsal pedis	Radial a.		Interarterial		Successful	
3	18	M	Degloving injury	Forearm	Groin		Cephalic v.	Intervenous	Congestion	Reexploration	Survival
4	38	M	Open fracture	Lower extremity	Groin		Saphenous v.	Intervenous		Successful	
5	34	F	Open fracture	Lower extremity	ALT	Ant. tibial a.	Ant. tibial v.	Interarterial and intervenous		Successful	
6	71	M	Hypopharyngeal ca.	Neck	Jejunum	Lingual a.	Internal jugular v.	Interarterial and intervenous		Successful	
7	22	M	Open fracture	Lower extremity	ALT	Post. tibial a.		Interarterial		Successful	
8	21	M	Open fracture	Lower extremity	ALT	Ant. tibial a.		Interarterial		Successful	
9	65	M	Osteomyelitis	Lower extremity	Fibula	Post. tibial a.	Post. tibial v.	Interarterial and intervenous		Successful	
10	59	M	Hypopharyngeal ca.	Neck	Jejunum	Lingual a.		Interarterial		Successful	
11	70	M	Osteomyelitis	Lower extremity	Fibula	Post. tibial a.	Post. tibial v.	Interarterial and intervenous		Successful	
12	57	M	Osteomyelitis	Lower extremity	Fibula	Post. tibial a.	Post. tibial v.	Interarterial and intervenous		Successful	
13	30	F	Open fracture	Lower extremity	Latissimus dorsi	Popliteal a.		Interarterial		Successful	
14	52	M	Osteomyelitis	Lower extremity	Fibula	Post. tibial a.	Post. tibial v.	Interarterial and intervenous	Congestion	Reexploration	Survival
15	84	M	Hypopharyngeal ca.	Neck	Jejunum	Lingual a.		Interarterial		Successful	

ALT: anterolateral thigh.

## References

[1] Whitney T. M., Buncke H. J., Lineaweaver W. C., Alpert B. S. (1989). Multiple microvascular transplants: a preliminary report of simultaneous versus sequential reconstruction. *Annals of Plastic Surgery*.

[2] Khouri R. K., Shaw W. W. (1989). Reconstruction of the lower extremity with microvascular free flaps: a 10-year experience with 304 consecutive cases. *Journal of Trauma*.

[3] Oliva A. (1993). Interposition vein grafting in head and neck reconstructive microsurgery. *Journal of Reconstructive Microsurgery*.

[4] Miller M. J., Schusterman M. A., Reece G. P., Kroll S. S. (1993). Interposition vein grafting in head and neck reconstructive microsurgery. *Journal of Reconstructive Microsurgery*.

[5] Germann G., Steinau H.-U. (1996). The clinical reliability of vein grafts in free-flap transfer. *Journal of Reconstructive Microsurgery*.

[6] Bayramiçli M., Tetik C., Sönmez A., Gürünlüoğlu R., Baltaci F. (2002). Reliability of primary vein grafts in lower extremity free tissue transfers. *Annals of Plastic Surgery*.

[7] Buncke H. J., Alpert B., Shah K. G. (1978). Microvascular grafting. *Clinics in Plastic Surgery*.

[8] Moneim M. S., Chacon N. E. (1985). Salvage of replanted parts of the upper extremity. *The Journal of Bone and Joint Surgery Series A*.

[9] Nahai F., Hagerty R. (1986). One-stage microvascular transfer of a latissimus flap to the sacrum using vein grafts. *Plastic and Reconstructive Surgery*.

[10] Hallock G. G. (1988). The interposition arteriovenous loop revisited. *Journal of Reconstructive Microsurgery*.

[11] Grotting J. C. (1991). Prevention of complications and correction of postoperative problems in microsurgery of the lower extremity. *Clinics in Plastic Surgery*.

[12] Horng S.-Y., Chen M.-T. (1993). Reversed cephalic vein: a lifeboat in head and neck free-flap reconstruction. *Plastic and Reconstructive Surgery*.

[13] Schusterman M. A., Miller M. J., Reece G. P. (1994). A single center’s experience with 308 free flaps for repair of head and neck cancer defects. *Plastic and Reconstructive Surgery*.

[14] Zhang F., Oliva A., Kao S. D., Newlin L., Buncke H. J., Daniller A. (1994). Microvascular vein-graft patency in the rat model. *Journal of Reconstructive Microsurgery*.

[15] Zhang F., Oliva A., Kao S. D., Newlin L., Buncke H. J. (1994). Microvascular vein grafts in the rat cutaneous free-flap model. *Journal of Reconstructive Microsurgery*.

[16] Kruse A. L. D., Luebbers H. T., Grätz K. W., Obwegeser J. A. (2010). Factors influencing survival of free-flap in reconstruction for cancer of the head and neck: a literature review. *Microsurgery*.

[17] Cheng H. T., Lin F. Y., Chang S. C. N. (2012). Evidence-based analysis of vein graft interposition in head and neck free flap reconstruction. *Plastic and Reconstructive Surgery*.

